# Ablation of CRBN induces loss of type I collagen and SCH in mouse skin by fibroblast senescence via the p38 MAPK pathway

**DOI:** 10.18632/aging.202744

**Published:** 2021-03-03

**Authors:** Seungje Jeon, Yi-Seul Yoon, Hyoung Kyu Kim, Jin Han, Kwang Min Lee, Jung Eun Seol, Steve K. Cho, Chul-Seung Park

**Affiliations:** 1 School of Life Sciences, Gwangju Institute of Science and Technology (GIST), Gwangju, Republic of Korea; 2 Integrated Institute of Bio-Medical Research, Gwangju Institute of Science and Technology (GIST), Gwangju, Republic of Korea; 3 Department of Physiology, BK21 Plus Project Team, College of Medicine, Smart Marine Therapeutics Center, Cardiovascular and Metabolic Disease Center, Inje University, Busan, Republic of Korea; 4 Department of Life Science and Environmental Biochemistry, and Life and Industry Convergence Research Institute, Pusan National University, Miryang, Republic of Korea; 5 Department of Dermatology, Inje University Busan Paik Hospital, College of Medicine, Smart Marine Therapeutics Center, Cardiovascular and Metabolic Disease Center, Inje University, Busan, Republic of Korea

**Keywords:** CRBN, senescence, collagen1, p38, SA-beta-Gal

## Abstract

Cereblon (CRBN) is a substrate receptor of the cullin-RING E3 ubiquitin ligase (CRL) complex that mediates the ubiquitination of several substrates. In this study, CRBN knockout (KO) mice exhibited decreased levels of stratum corneum hydration (SCH) and collagen I expression with an elevated protein level of matrix metalloprotease 1 (MMP1). The absence of cereblon in the skin of CRBN KO mice mimics the damage caused by narrowband ultraviolet B (NB-UVB). The primary CRBN deficient mouse embryonic fibroblasts (MEFs) undergo G2/M-arrested premature senescence via protein signaling of p38 MAPK and its dependent p53/p21pathway. The absence of CRBN induced the markers of cellular senescence, such as the senescence-associated heterochromatin foci (SAHF), SA-β-Gal staining, and p21 upregulation while the ectopic expression of CRBN reversed the phenotypes of SA-β-Gal staining and p21 upregulation. Reversion of the decreased protein level of collagen I was demonstrated after the reintroduction of the CRBN gene back into CRBN KO MEFs, validating the promising role of CRBN as a potential regulator for the function of the skin barrier and its cellular homeostasis.

## INTRODUCTION

Skin is the largest organ of the body [1] and functions as a physical barrier that regulates the flow of water and electrolytes while protecting against toxic substances [2]. The collagen fibers secreted from the fibroblast, which is the main cellular component of the dermis, plays an important role in the homeostasis and function of the skin [3]. The senescence of skin is closely related to the decrease in the dermal collagen content and the proliferation of skin fibroblasts [4]. Moreover, previous research revealed that senescent cells modulate their environment by secreting inflammatory cytokines, growth factors, and matrix metalloproteinases (MMPs), which are collectively known as senescence-associated secretory phenotypes (SASP) [5]. MMP1 is a family of zinc-dependent enzymes, which can specifically degrade the proteins in connective tissues such as collagen I [6]. Cellular senescence is a permanent form of cell cycle arrest [7] that occurs in primary cells after cell division while the cell remains metabolically and synthetically active [8]. Cellular growth arrest can be caused by various stresses, such as telomere uncapping, DNA damage, or oxidative stress caused by reactive oxygen species (ROS) [6]. Cellular senescence is characterized by increased levels of senescence-associated biomarkers, such as senescence-associated heterochromatin foci (SAHF) using HP1-γ as a marker, SA-β-Gal, reduced proliferative activity, and increased expression levels of senescence-associated genes, including cyclin-dependent kinase inhibitors, such as p16, p21, and p53 [9]. Several factors, including protein-signaling pathways, are involved in the regulation of p21. One of the major pathways to control the level of p21 is via p53, which is a transcription factor [10].

Cereblon (CRBN) was initially identified as a gene responsible for a mild form of autosomal recessive non-syndromic mental retardation (ARNSMR) in the human brain [11]. Since then, CRBN has been characterized in several distinct cellular contexts. CRBN has been reported as a primary target protein of immunomodulatory drugs (IMiDs), such as thalidomide, and also shown to be a substrate receptor, or DCAFs (DDB1 and CUL4-associated factors), of the cullin-RING E3 ubiquitin ligase (CRL) complex [12]. CRBN plays role in multiple biological processes by mediating the ubiquitination of target substrates [13]. Several target proteins of CRBN such as AMPK [14–16], GS [17], Casein Kinase1 [18], and BK channel [19] have been elucidated in previous studies.

CRBN is closely associated with cell-cycle proliferation and metabolism of both normal and tumor cells [13]. mRNA expression of CRBN varied in different cancer cells [20], and many somatic mutations in the CRBN gene were found in the database of cancer patients [21]. CRBN was also shown to determine brain size by regulating the proliferation of neural stem cells (NSCs) during development [22]. The silencing of CRBN impaired the ability of lenalidomide to induce p21 expression, indicating that lenalidomide directly inhibits CLL cell proliferation in a CRBN/p21-dependent manner [23]. Recently, p53 was reported to be a direct substrate of CRBN, yet subcellular distribution, protein expression level, and ubiquitination were not significantly affected [24]. In the interim, our research group reported that the permanent cell line of mouse embryonic fibroblast (MEF) that lacked CRBN was strongly resistant to hydrogen peroxide (H_2_O_2_), which is a typical ROS inducer for activating cellular senescence, and the loss of CRBN exhibited the pre-activation of p38 MAPK [25]. Despite the circumstantial evidence, CRBN's functional roles in cellular senescence and its mechanisms, along with any potential physiological manifestations, have not been rigorously investigated. In this study, CRBN knockout (KO) mice were found to exhibit decreased levels of stratum corneum hydration (SCH) and collagen I expression with an elevated protein level of matrix metalloprotease 1 (MMP1). The absence of cereblon in the dermis of CRBN KO mice mimics the damage caused by narrowband ultraviolet B (NB-UVB). The primary CRBN deficient mouse embryonic fibroblasts (MEFs) were also found to undergo G2/M-arrested premature senescence via protein signaling of p38 MAPK and its dependent p53/p21pathway.

## RESULTS

### The mice ablated CRBN gene exhibits decreased levels of skin hydration

**Figure 1:**
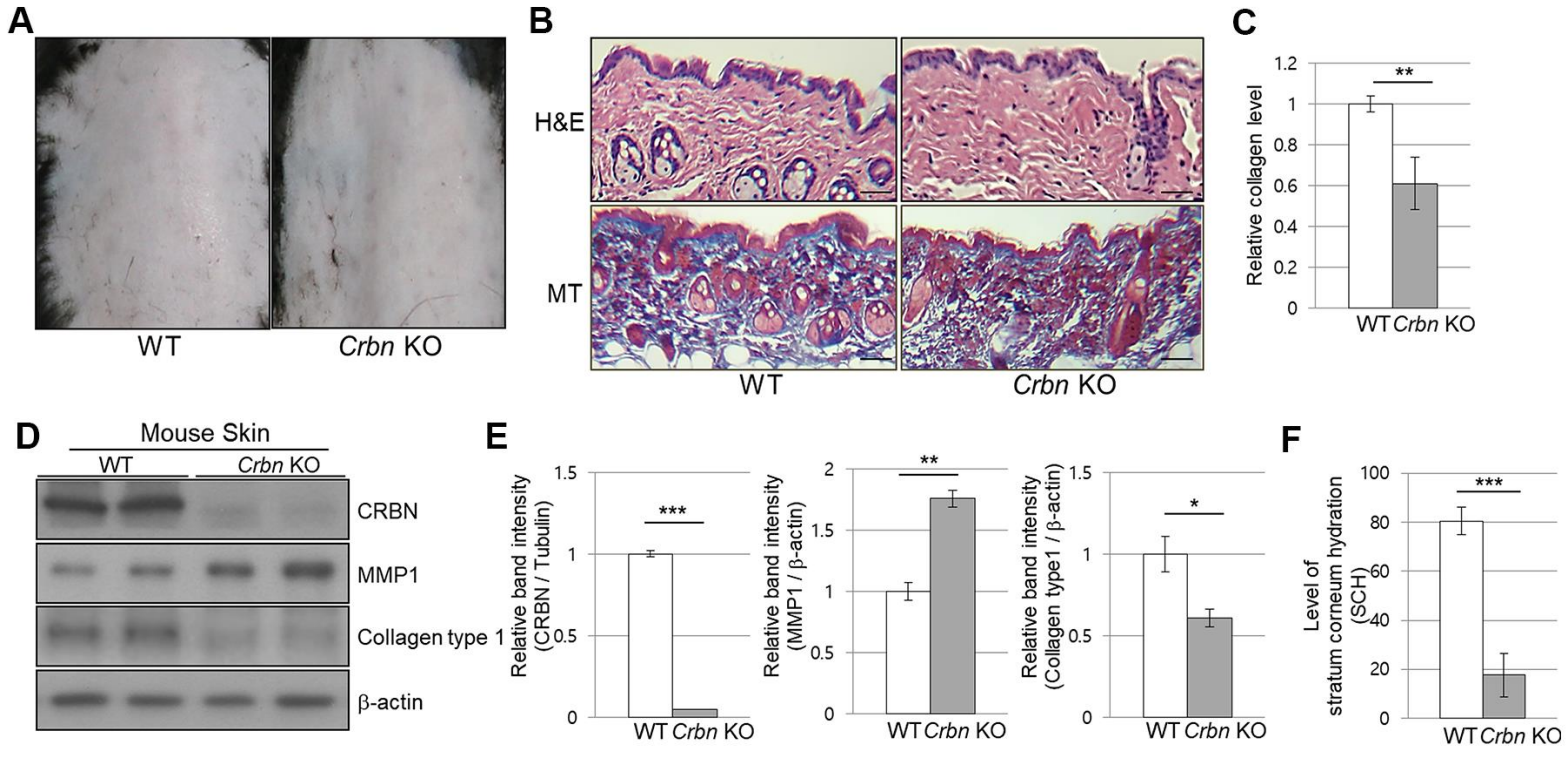
**The mice ablated CRBN gene exhibits decreased levels of skin hydration.** (**A**) The appearance of the back skin to compare the control WT and CRBN KO groups, respectively. (**B**) Relative initial Stratum Corneum Hydration (SCH) level in the skin of control WT and CRBN KO mouse skin. (**C**) Representative image of H&E and Masson’s trichrome stained section showing the structure of the epidermis and the dermis from the back skin of 12-weeks old female mice. (**D**) Analysis of the level of collagen fibers in the skin section in (**C**). (**E**) Western blots analysis using the protein lysate from the mouse skin. Proteins were subjected to immunoblotting using the anti-CRBN, anti-MMP1, anti-Collagen type1, and β-actin antibodies. The β-actin was used as a loading control. (**F**) Relative band intensities determined by densitometric-analysis of each protein in blot (**E**). The results shown are representative images of independent experiments (n=5). Scale bar = 100μm. *P < 0.05; **P < 0.01; ***P < 0.005; n.s., not significant.

When the CRBN KO mice were first examined, no apparent epidermal phenotypes such as hair loss or skin folding were observed from gross observations (Figure 1A). Haemotoxylin and Eosin (H&E) staining showed no significant changes in the morphology of the skin layers. However, Masson’s trichrome (MT) staining showed a significant decrease of collagen I secreted into the extracellular space (Figure 1B, 1C). Western blot analysis confirmed that the decreased expression of collagen I while matrix metalloproteinase 1 (MMP1) protein expression was elevated drastically in the skin of CRBN KO compared to that of WT mice (Figure 1D, 1E). To test the function of the skin barrier in CRBN KO mice, the water content in the uppermost layer of the skin was measured as shown in Figure 1F. Measurement of stratum corneum hydration (SCH) is a hallmark of skin abnormalities, and the SCH level in CRBN KO mice decreased by more than 3 times (Figure 1F). Taken together, impaired skin barrier function due to stratum corneum (SC) abnormalities and abnormal protein expression patterns were observed in CRBN ablated mice skin.

### The absence of CRBN in mice skin mimics the UV-damaged phenotype

**Figure 2:**
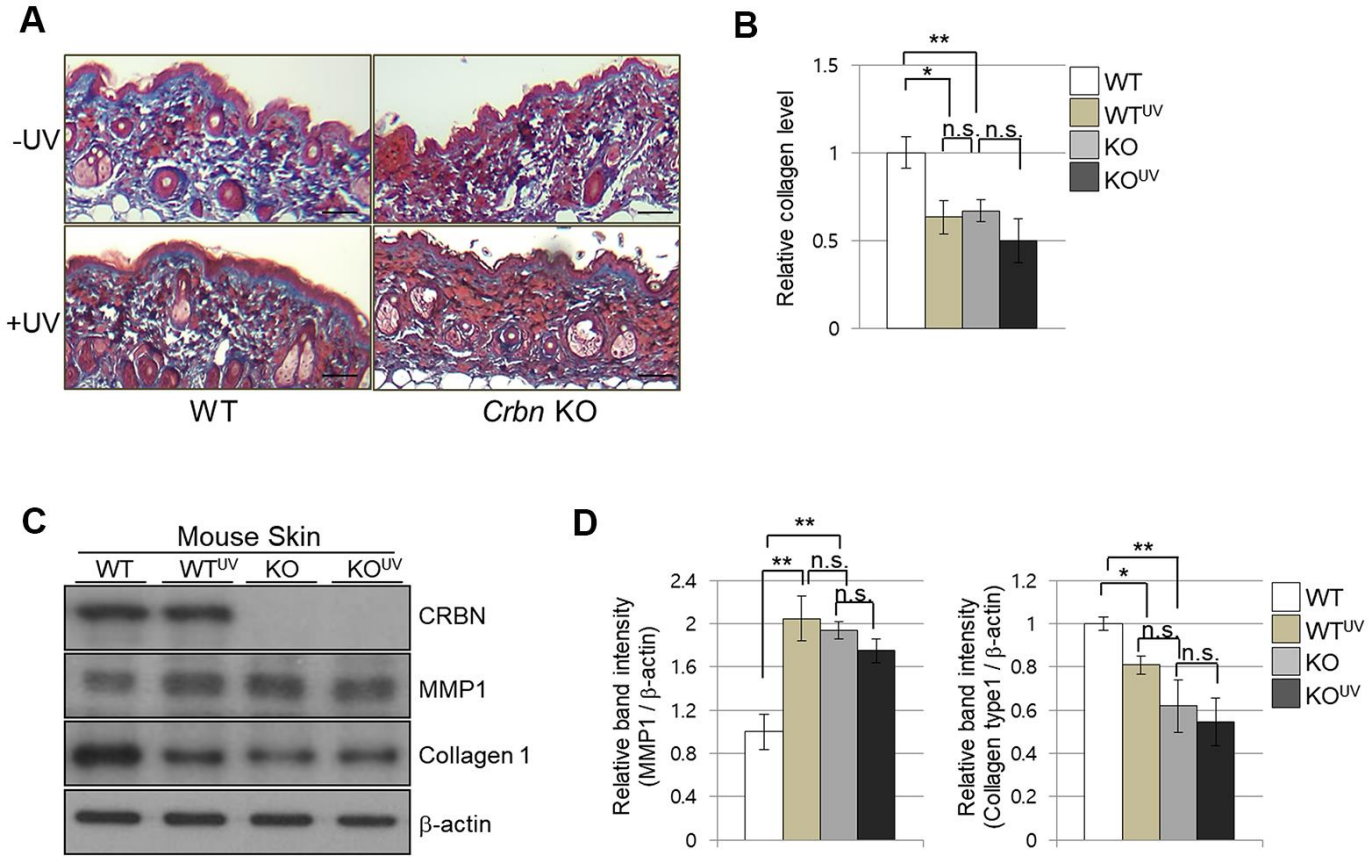
**The absence of CRBN in mice skin mimics UV-damaged phenotype.** (**A**) Representative image of Masson’s trichrome stained section showing the skin structure affected by ultraviolet irradiation after 4 weeks. (**B**) Analysis of the level of collagen fibers in the skin section in (**A**). (**C**) Western blots analysis using the protein lysate from the mouse skin. Proteins were subjected to immunoblotting using the anti-CRBN, anti-MMP1, anti-Collagen type1, and β -actin antibodies. The β-actin was used as a loading control. (**D**) Relative band intensities determined by densitometric-analysis of each protein in blot (**C**). The results shown are representative images of independent experiments (n=5). Scale bar = 100μm. *P < 0.05; **P < 0.01; ***P < 0.005; n.s., not significant.

To further explore the abnormalities of CRBN deficient mouse skin, both WT and CRBN mice were exposed to 311 nm narrowband ultraviolet B (NB-UVB) according to standard protocols with a mean dose of 10J/m^2^ for 3 min. Histopathological examination revealed that the NB-UVB induced significant damage on the dermal collagen layer of WT mice as shown in Figure 2A (*left* panel) while the dermis of CRBN mice was not further damaged by the NB-UVB (Figure 2B, *right* panel). The abnormal collagen layer of dermis induced by the NB-UVB in the WT mice was further confirmed by immunoblot analysis as shown in Figure 2C, 2D. After a close comparison, CRBN deficient dermis resembled that of NB-UVB treated skin.

### The absence of CRBN induces the senescence markers of cellular senescence in the primary mouse embryonic fibroblasts

**Figure 3:**
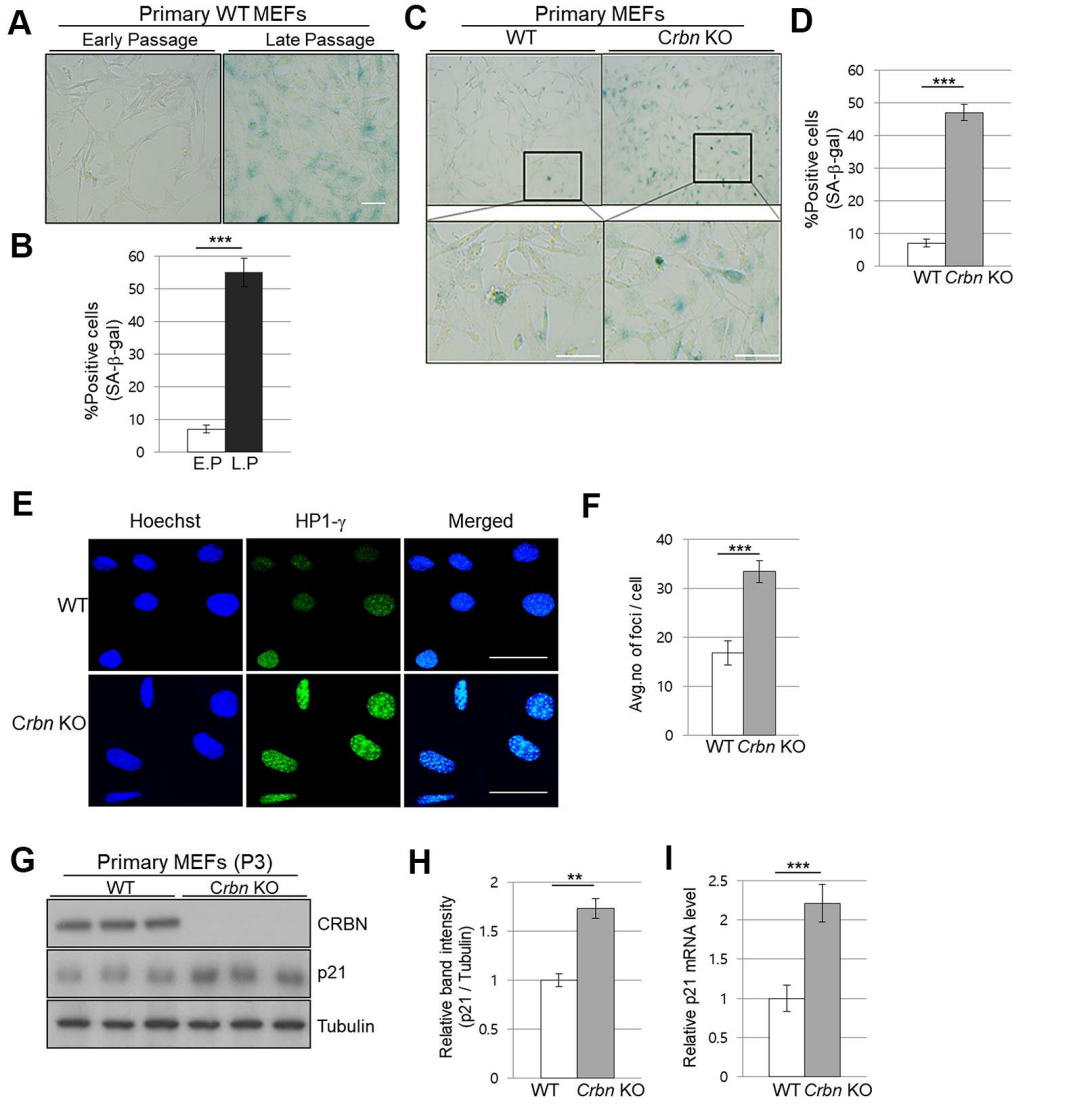
**The absence of CRBN induces the markers of cellular senescence in the primary mouse embryonic fibroblast.** (**A**, **C**) Representative staining images showing SA-β-Gal(blue-stained cells) in primary MEFs. E.P = early passage (P3), L.P = late passage (P8-10) Scale bar = 100μm. (**B**, **D**) Quantification of SA-β-Gal-positive cells shown in (**A**, **C**) respectively. Results are expressed as the percentage of stained cells (mean ± SEM). (**E**) Representative images for HP1-γ foci by immunofluorescence staining. (**F**) Quantitative analysis of HP1-γ foci per cell. (**G**) Endogenous levels proteins as determined by western blot analysis using extracts from the WT and CRBN KO MEFs. The passage numbers were indicated in the figure. Proteins were subjected to immunoblotting with the anti–CRBN, anti-p21, and anti–Tubulin antibodies. Tubulin was used as a loading control. (**H**) The relative band ratio of CRBN and p21 to tubulin as determined by densitometric analysis of the blots in (**G**). (**I**) Total RNA was isolated from each type of MEFs and subjected to qRT-PCR to measure the mRNA expression of p21. Expression was normalized against β-actin mRNA levels. Fold changes in the mRNA levels relative to control WT MEF is shown. The results shown are representative of five independent experiments. *P < 0.05; **P < 0.01; ***P < 0.005; n.s., not significant.

To assess the physiological relevance of our findings, primary mouse embryonic fibroblasts (MEFs) from WT and CRBN KO mice were used to analyze the degree of cellular senescence by the senescence-associated beta-galactosidase (SA-β-Gal) staining. As shown in Figure 3A, 3B, the typical SA-β-Gal positive signal started visualizing after passage number 8 in WT MEFs. However, ~40% of CRBN KO MEFs showed significant SA-β-Gal signaling as early as passage number 3, indicating potential premature cellular senescence in the CRBN KO MEFs (Figure 3C, 3D). In determining whether the senescence-associated heterochromatin foci (SAHF) increased with CRBN KO, the primary MEFs were assessed by using immunofluorescence (IF) staining with HP1-γ, which is a molecular marker for SAHF. These results confirmed that the average number of foci per cell and deposition of HP1-γ increased significantly in CRBN KO MEFs compared to that of WT (Figure 3E, 3F). The elevated protein expression of HP1-γ in CRBN KO MEFs was also confirmed by Western blot (Supplementary Figure 1). Next, mRNA and protein expression of p21, a cellular senescence marker, were analyzed by qRT-PCR and Western blot. Both mRNA and protein expressions of p21 were elevated drastically in the CRBN deficient MEFs (Figure 3G–3I), suggesting that CRBN has a role in arresting cell cycle progression.

### CRBN deficient fibroblast exhibits G2/M cell cycle arrest

**Figure 4:**
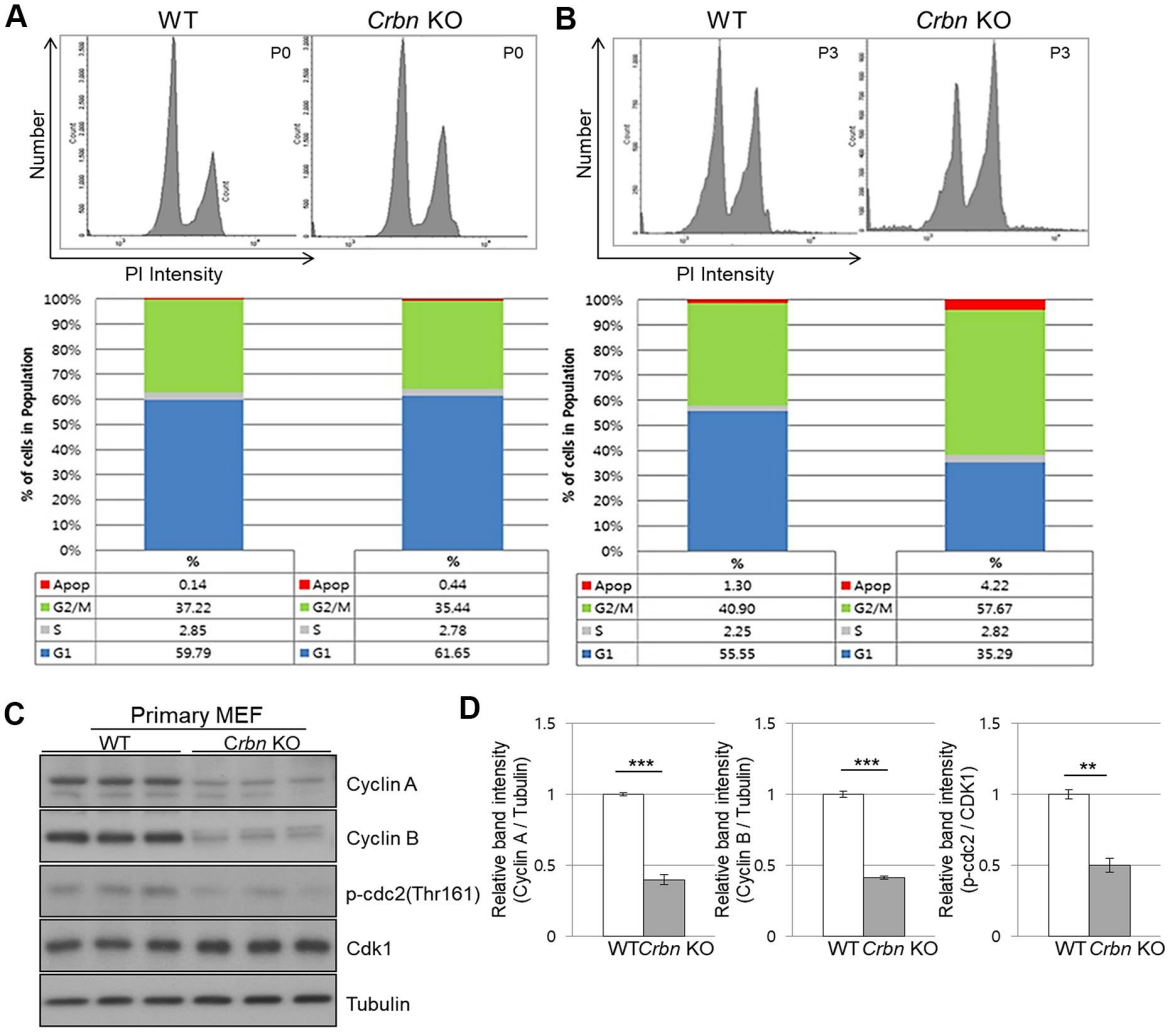
**CRBN deficient fibroblast exhibits G2/M cell cycle arrest.** (**A**, **B**) PI staining and cell cycle distribution analysis of WT and CRBN KO Primary MEFs in P0 and P3. Cells were stained with PI and analyzed for cell cycle distribution using flow cytometry. Representative images of flow cytometry plots are shown. The graph indicates the distribution of each cell cycle phase with different colors, G0/G1(blue), S(gray), and G2/M(green) phases. (**C**) Western blots analysis using extracts of MEF cells in the early passage were immunoblotted with the anti-Cyclin A, anti-Cyclin B, anti-Cdk1, anti-p-cdc2, and anti–Tubulin antibodies. Tubulin was used to confirm equal protein loading. (**D**) Relative band intensities as determined by densitometric analysis of the blots in (**C**). The results shown are representative of five independent experiments. *P < 0.05; **P < 0.01; ***P < 0.005; n.s., not significant.

To further explore the role of CRBN in the cell cycle, both primary WT and CRBN KO MEFs were treated with propidium iodide (PI) staining and then subjected to flow cytometry. While the distribution of the cell cycle phase was shown to be typically normal in the initial passage (P0) of each fibroblast (Figure 4A), the portion of the G2/M phase was accumulated as early as passage number 3 (P3) of CRBN deficient fibroblasts (Figure 4B). The percentage distributions of cell cycle phases of WT and CRBN deficient cells in (P0) and (P3) are shown in each figure. Western blot analysis of proteins involved in the cell cycle revealed that protein expressions of cyclin A, cyclin B, and the phosphorylation of CDK1 (cyclin-dependent kinase 1) were significantly decreased in CRBN deficient fibroblasts compared to those of WT cells (Figure 4C, 4D). Taken together, these results suggest the role of CRBN during premature senescence in CRBN deficient MEFs at the G2/M phase.

### The absence of CRBN activated the p38 MAPK/p53 signaling axis resulting in p21 upregulation

**Figure 5:**
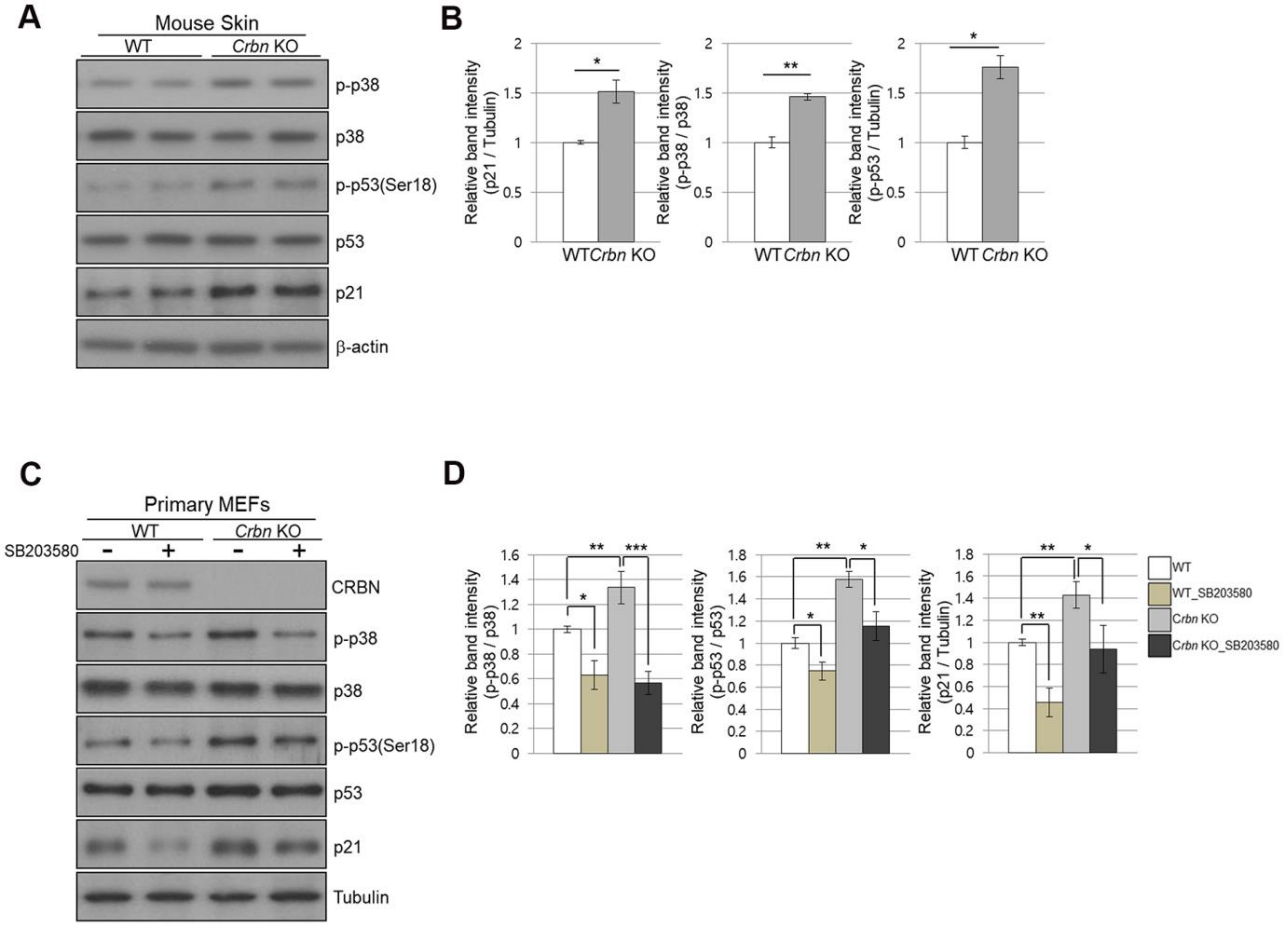
**The absence of CRBN activated the p38 MAPK/p53 signaling axis, resulting in p21 upregulation.** (**A**) Endogenous levels proteins as determined by western blot analysis using extracts from mice skin. Proteins were subjected to immunoblotting using the anti-CRBN, anti-p21, anti-p38, anti-p-p38, anti-p53, anti-p-p53(Ser18), and anti–Tubulin antibodies. (**B**) Relative band intensities determined by densitometric analysis of each protein in blot (**A**). (**C**) Western blots analysis using protein lysate from the WT and CRBN KO MEFs. 10mM of SB203580 was treated to each type of cell for 2hr. Proteins were subjected to immunoblotting with the anti-CRBN, anti-p38, anti-p-p38, anti-p53, anti-p-p53(Ser18), anti-p21, and anti–Tubulin antibodies. The tubulin was used as a loading control. (**D**) The relative band ratio as determined by densitometric analysis of the blots in (**C**). The results shown are the means ± SEM of five independent experiments *P < 0.05; **P < 0.01; ***P < 0.005; n.s., not significant.

Next, we investigate whether CRBN deficiency affects p53/p21 and p38 MAPK signaling pathways. Figure 5A, 5B showed significant activation of p38 MAPK and p53, which were confirmed in the skin of CRBN KO mice by immunoblotting, suggesting that activation of p38 induces transcriptional activity of p53 and p21. To further explore whether p38 affects p53 and p21 functions, SB203580, which is a p38 specific inhibitor, was used. As shown in Figure 5C, 5D, the treatment of the MEFs with SB203580 prevented the activation of p38 and p53 with decreased protein expression of p21 in the CRBN deficient MEFs. Overall, the results confirmed that CRBN depletion activated the p38 MAPK/p53 signaling axis, resulting in p21 upregulation.

### Ectopic expression of CRBN reversed the premature senescence phenotype and downregulation of collagen I protein expression in CRBN KO MEFs

**Figure 6:**
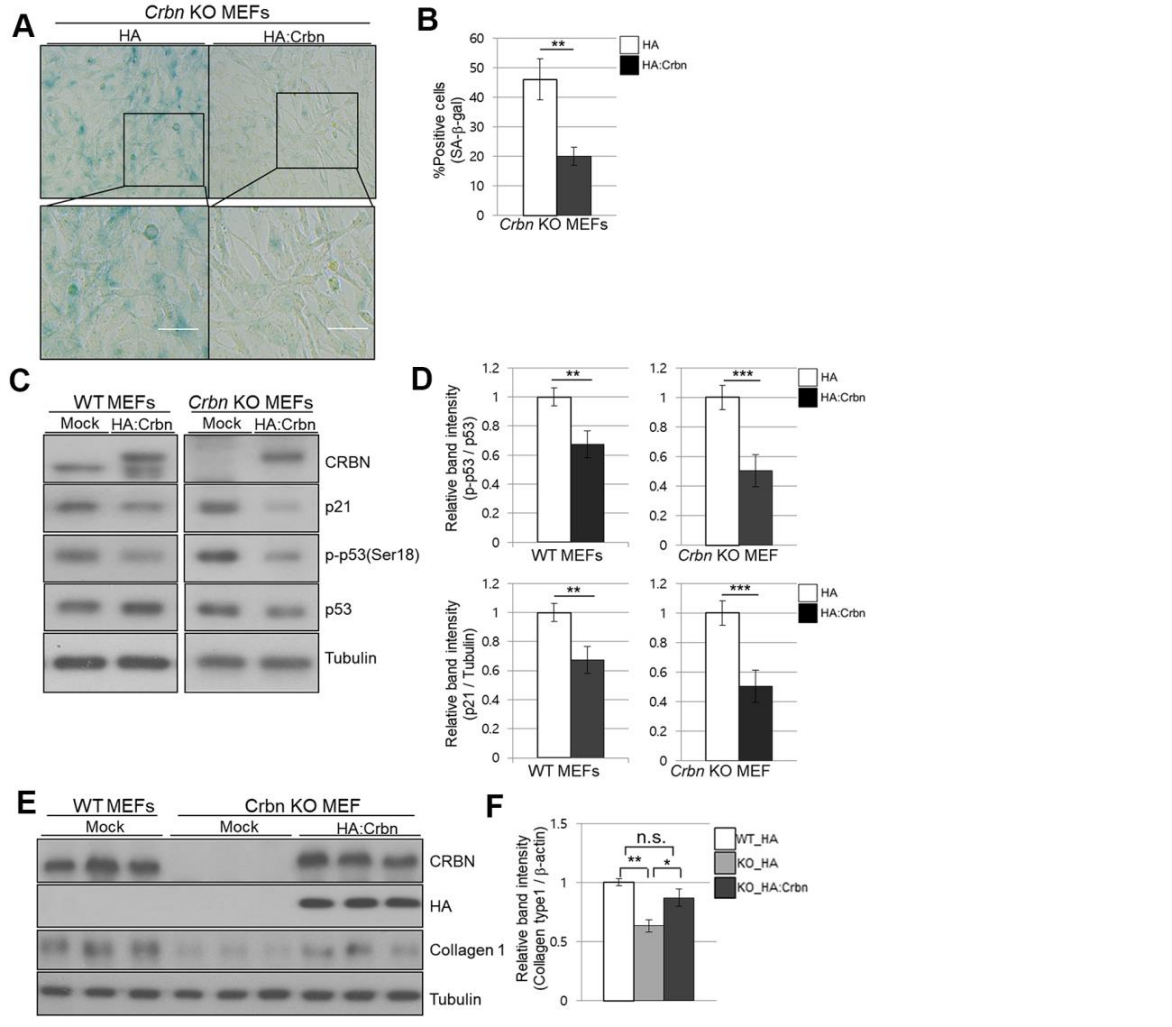
**Ectopic overexpression of CRBN clears the SA-β-Gal signal and recovers the protein level of Collagen I in cultured fibroblast.** (**A**) Representative staining images showing SA-β-Gal (blue-stained cells) along with the overexpression of CRBN in primary MEFs. Scale bar = 100μm. (**B**) Quantification of SA-β-Gal-positive cells shown in (**A**). Results are expressed as the percentage of stained cells (mean+SEM). The results shown are representative of four independent experiments. (**C**) The WT and CRBN KO primary MEFs were transiently transfected with HA: CRBN or empty vector. Cells were harvested after 24h and the protein lysates were subjected to immunoblotting with the anti-CRBN, anti-p53, anti-p-p53(Ser18), anti-p21, and anti–Tubulin antibodies. (**D**) The relative band ratio as determined by densitometric analysis of the blots in (**C**). (**E**) Cell lysates were prepared from WT and CRBN KO primary MEFs transfected with HA: CRBN or empty vector. Western blots of the protein lysate were probed with the anti-CRBN, anti-HA, anti-collagen I, and anti–Tubulin antibodies. Tubulin was used for equal protein loading. (**F**) The relative band intensity was measured by densitometric-analysis of the blots in (**E**). The results shown are representative of five independent experiments. *P < 0.05; **P < 0.01; ***P < 0.005; n.s., not significant.

To confirm the role of CRBN in premature senescence in MEFs stained by SA-β-Gal and upregulation of p21 via p38 MAPK/p53 pathways, ectopic expression of CRBN was performed in CRBN KO MEFs. As shown in Figure 6A, 6B, SA-β-Gal staining was significantly reduced compared to mock-transfected CRBN MEFs. The upregulation of p21 and p53 activation were reversed after the reintroduction of CRBN back into CRBN MEFs, SHSY-5Y and HEK293T cells (Figure 6C, 6D and Supplementary Figure 2). The downregulation of collagen I protein was also reversed via ectopic expression of CRBN in CRBN KO MEFs, confirming the role of CRBN in abnormal collagen layer formation in CRBN KO dermis (Figure 6E).

## DISCUSSION

Cereblon (CRBN) is a substrate receptor of the cullin-RING E3 ubiquitin ligase (CRL) complex that mediates the ubiquitination of several substrates. However, previous studies have not revealed a potential link between CRBN and stratum corneum (SC), which maintains permeability protection from uncontrolled water and electrolyte loss as well as being critical for temperature regulation [26]. In this study, a new role of CRBN as a potential regulator for the function of the skin barrier and its cellular homeostasis was demonstrated. A review of the literature suggests that our study may be the first to implicate CRBN's functional role in skin homeostasis by showing decreased levels of stratum corneum hydration (SCH) and collagen I expression with an elevated protein level of matrix metalloprotease 1 (MMP1) in CRBN knockout (KO) mice.

Because CRBN is a substrate receptor for CRL4 E3 ubiquitin ligase, it is conceivable that the loss of CRBN failed to recruit its endogenous substrate(s) that are important for cellular homeostasis in maintaining the skin barrier function. We have found that the SCH level in CRBN KO mice decreased by more than 3 times as adequate SC hydration is required for maintaining skin plasticity and barrier integrity.

The skin is also continuously exposed to UV rays, resulting in premature skin aging, and to pro-oxidative environments such as oxidative stress, which is an imbalance between the formation of cellular oxidants and the anti-oxidant defense systems [27]. We have demonstrated that NB-UVB significantly damaged the collagen layer of the dermis in WT mice and, after a close comparison, found that CRBN deficient dermis resembled that of NB-UVB treated skin.

We have also found that more than 40% of CRBN KO MEFs showed significant SA-β-Gal staining, as well as increased expression of the HP1-γ which is a marker of senescence-associated heterochromatin foci (SAHF) as early as passage number 3 indicating potential premature cellular senescence in the CRBN KO MEFs. The CRBN deficient cells induced the upregulation of p21; however, p21 is not a substrate of CRBN (data not shown).

Cell cycle checkpoints at the G2/M is critical for maintaining and regulating the cell division through the cell cycle. The portion of the G2/M phase increased as early as passage number 3 (P3) of CRBN deficient fibroblasts when compared to that of WT cells (57% from 40%). Levels of cyclin A and B as well as the phosphorylation of CDK1 reduced significantly in CRBN deficient fibroblasts when compared to those of WT cells, suggesting premature senescence at the G2/M phase.

We have also shown activation of p38 mitogen-activated protein kinase (p38 MAPK) and its dependent downstream of p53 and the upregulation of p21 in the CRBN deficient MEFs. Although there are still unanswered questions for the underlying mechanisms to explain the relationship between CRBN and p38 MAPK activation, the reactive oxygen species (ROS) probably is one of the key upstream activators of the p38 MAPK signaling pathway [28]. CRBN-deficient primary fibroblasts were resistant to oxidative stress evoked by exogenous oxidants such as H_2_O_2_ or UV (data not shown). Because oxidative stress is known to affect cellular senescence, CRBN could be involved in cellular senescence by intracellular ROS with key regulatory enzymes contributing to the cellular redox homeostasis. Nevertheless, the regulation of cellular oxidative status by CRBN is a tempting proposition that requires further investigation.

CRBN KO mice have been characterized in multiple phenotypical contexts. A series of studies elucidating the physiological function of CRBN has also been reported in various disease models or under specific challenges [15, 24, 29–31]. The deficiency of CRBN has exhibited protective effects or positive influence under various adverse challenges *in vivo*. For example, the loss of the CRBN gene protected mice from obesity, fatty liver, and insulin resistance induced by a high-fat diet [15]. CRBN KO mice attenuated myocardial ischemia-reperfusion injury [32] as well as ameliorated alcoholic liver disease [33].

Finally, the ectopic expression of CRBN in CRBN KO MEFs has reversed premature senescence phenotypes such as SA-β-Gal staining and the upregulation of p21 via p53 activation. We have also analyzed the interaction between p38, p53, and p21 by inhibiting p38 with a specific pharmacological inhibitor and by evaluating p53 phosphorylation and consequent downregulation of p21 in the MEFs. SB203580 notably decreased p38 and p53 phosphorylation resulting in the downregulation of p21.

In summary, the loss of the CRBN gene exhibited premature senescent phenotypes in both skin and the primary fibroblast. CRBN KO mice showed a reduced level of hydration and collagen expression in the skin. We found that a higher percentage of the cells were arrested at G2/M and the expression levels of cell cycle checkpoint proteins were significantly altered. The ablation of CRBN activated the signaling pathway involving p38 MAPK and p53, resulting in the upregulation of p21. The data obtained from this study facilitate an understanding that the role of CRBN as a potential regulator for the function of the skin barrier, and its cellular homeostasis may be valuable in providing novel clinical applications for disorders associated with premature senescence.

## MATERIALS AND METHODS

### Experimental animals

C57BL/6J WT mice and CRBN-KO mice [15] (4-9 weeks, female) were housed in a room with a standard chow diet and water ad libitum in specific pathogen-free conditions (IVC-system) with a 12-h light-dark cycle. All materials for the maintenance of animals were provided by the Gwangju Institute of Science and Technology Animal Care and Use Committee. All animal experiments were conducted according to the institutional guidelines of the Gwangju Institute of Science and Technology (GIST).

### Histological analysis of the skin

Each type of mice was sacrificed, and the skin tissues were fixed in 10% neutral buffered formaldehyde and embedded in paraffin. Paraffin sections (5 mm) were then subjected to hematoxylin-eosin (H&E) and Masson’s Trichrome (MT) staining. The colored images were processed using ImageJ (Wyne Rasband, National Institutes of Health, Bethesda, USA).

### UV irradiation

At the end of the 4 weeks, each type of mice was exposed to UV-irradiation to cause skin damage at a fixed time every day. The type of lamp used is narrow-band UVB (Philips TL20W-01RS). Before each irradiation, UV power was measured using a radiometer. After measuring, the UV emission time is to be calculated to radiate a constant amount of UV light.

### Skin hydration evaluation

The measurement of the skin hydration content was carried out using a probe (Corneometer and Cutometer MPA580, Courage and Khazaka Kő in Germany) under standard conditions of temperature and humidity (T° = 20-22° C, humidity 40-60%). The spring-loaded probe in the head of the Corneometer was gently stuck to the back skin of mice for measurements. The higher the measured value, the higher the moisture content of the skin’s surface.

### Cell culture

The primary mouse embryonic fibroblasts (MEFs) were cultured in Dulbecco's modified Eagle's medium (DMEM, Hyclone) with 10% (v/v) fetal bovine serum (FBS, Hyclone). The WT and Crbn (-/-) primary MEFs were isolated from E13.5-14.5 embryos born to heterozygous intercrosses and genotyping was followed as previously described [15]. Torsos from each type of embryo were washed and minced in 2 ml PBS using a syringe and 18-gauge needle. After removing large fragments, the suspension was placed in a culture dish. After stabilization, each type of primary MEFs was assayed at passages within 3–10.

### Senescence-associated β-galactosidase staining

MEF cells were plated at a density of 1 × 10^5^ cells in 35 mm dishes. Cells were fixed and stained following the manufacturer’s recommendations supplied by the Senescence β-Galactosidase Staining Kit (9860; Cell Signaling Technology, Danvers, MA, USA). The population of SA-β -Gal positive cells was determined by counting 400 cells in at least 5 fields per dish, and images were taken using a phase-contrast microscope at 400× magnification (Olympus, Japan). The proportions of cells positive for SA-β -Gal activity are shown as the percentage of the total number of cells counted in each dish.

### Immunofluorescence (IF) staining

MEF cells were plated in 12-well plates with coverglass for imaging. The MEF cells were fixed in 4% PFA and permeabilized in 5% Triton X-100–PBS. The cells were incubated with HP1-γ polyclonal antibody antibodies (1 : 250, Abcam-ab213167) overnight at 4° C. After washing twice, cells were incubated with the Alexa-conjugated secondary antibodies for 1 hour. The cells were also counterstained with Hoechst dye solution before mounting for the nuclei. The slides were observed in a laser confocal microscope at 600x magnification. Olympus Fluoview Viewer was used to image and quantify the fluorescence.

### PI staining

For DNA content analysis, the cells were harvested from 6-well plates by trypsinization, rinsed with PBS, fixed in ice with 70% ethanol for 15min. The cells were then centrifuged at 1,000 × g for 5 min at 4° C and washed with PBS. After centrifugation at 500 × g for 10 min at 4° C, the cells were resuspended in a pre-mix solution containing propidium iodide (Sigma-Aldrich, p4864), RNase A, and Triton® X-100. After 30-min incubation in the dark incubator, the stained cells were analyzed on a FACSCanto II (BD Biosciences) or FACSCalibur (BD Biosciences) flow cytometer. Data were collected using a FACSCanto II (BD Biosciences) and analyzed with FlowJo software.

### Western blotting

For protein preparation, the mice's skin and cells were lysed with a 1:10 ratio of Tris-Cl buffer (20 mM Tris-Cl [pH 7.4], 0.32 M sucrose, 1 mM EDTA, 1 mM EGTA) using a homogenizer (Thomas Scientific). Protein samples were boiled with 2X sample buffer (24 mM Tris-Cl [pH 6.8], 10% Glycerol, 0.04% Bromophenol blue, 0.8% SDS) to produce samples for Western blot analysis. Proteins were separated in 6-10% SDS-PAGE gels at 80-90 V in the stacking gel and 140-150 V in the running gel until the target protein reached the medial part of the gel. The proteins were subsequently transferred to a PVDF membrane (GE Healthcare Life Sciences, #10600021). Membranes were blocked with 3% BSA prepared in 1X TBS-T (10 mM Tris-HCl, 100 mM NaCl, and 0.2% Tween 20, pH 7.5) for 50 min. The blots were then incubated with the following primary antibodies: CRBN (HPA 045910), MMP 1 (GTX100534), Collagen type 1 (ABT123), p21 (ab188224), Cyclin A (PA5-36048), Cyclin B (CST #4138), p-cdc2 (CST #9111), Cdk1 (Invitrogen, # 33-1800), p38 (CST #9212), p-p38 (CST #9211), p53 (CST #2524), p-p53 (CST #9284), HA (Invitrogen, # 26183), beta-actin (CST #4967), Tubulin (CST #2144). After washing twice in 1X TBS-T for 15 min, the membranes were incubated with peroxidase-conjugated anti-rabbit IgG (Jackson ImmunoResearch Laboratories, #111-035-003) or anti-mouse IgG (Jackson Immuno Research Laboratories, #115-035-003) for 50 min at room temperature. After washing twice in 1X TBS-T for 15 min, proteins were developed using enhanced chemiluminescence detection reagent (GE Healthcare Life Sciences, #RPN2209).

### Quantitative real-time PCR analysis

Total RNA was isolated from wild-type and CRBN−/− MEFs by TRIzol reagent (Invitrogen), according to the manufacturer's protocol. Complementary DNA (cDNA) was synthesized using CycleScript RT PreMix (Bioneer). mRNA levels were measured using TB Green™ Premix Ex Taq™ (TaKaRa) and Thermal Cycler Dice Real-Time System. The following primers were used for amplification. p21, forward: 5′ AAT CCT GGT GAT GTC CGA CC -3′, and reverse: 5′-AAA GTT CCA CCG TTC TCG G-3′; 18s rRNA, forward: 5′-GTA ACC CGT TGA ACC CCA TT-3′, and reverse: 5′-CCA TCC AAT CGG TAG TAG CG-3′. Expression was normalized to 18 s rRNA levels.

### Plasmid construction and transfection

HA-tagged CRBN were generated as described previously [14]. Cells were transfected using either Lipofectamine2000 (Invitrogen) or FuGENE HD (Promega) according to the manufacturer's protocols.

### Statistical analysis

Data were quantified using ImageJ and graphs were produced with Microsoft Office PowerPoint 2013 and Origin 9.1. All displayed data are expressed as mean ± SEM. The significant differences between groups were determined by a two-tailed unpaired Student’s t-test for most of the experiments except the analysis of SCH (Mann-Whitney U-test was used to analyze the difference of the average values obtained by the two groups). Differences with p < 0.05 were considered statistically significant.

### Data availability statement

No datasets were generated or analyzed during the current study.

## Supplementary Material

Supplementary Figures
