# Supplementary material for: Ablation of CRBN induces loss of type I collagen and SCH in mouse skin by fibroblast senescence via the p38 MAPK pathway
Source: Aging (Albany NY). 2021 Mar 3;13(5):6406–19. doi: 10.18632/aging.202744 (PMC7993720; doi:10.18632/aging.202744)
Supplement: Supplementary Figures [file aging-13-202744-s001.pdf]

SUPPLEMENTARY FIGURES

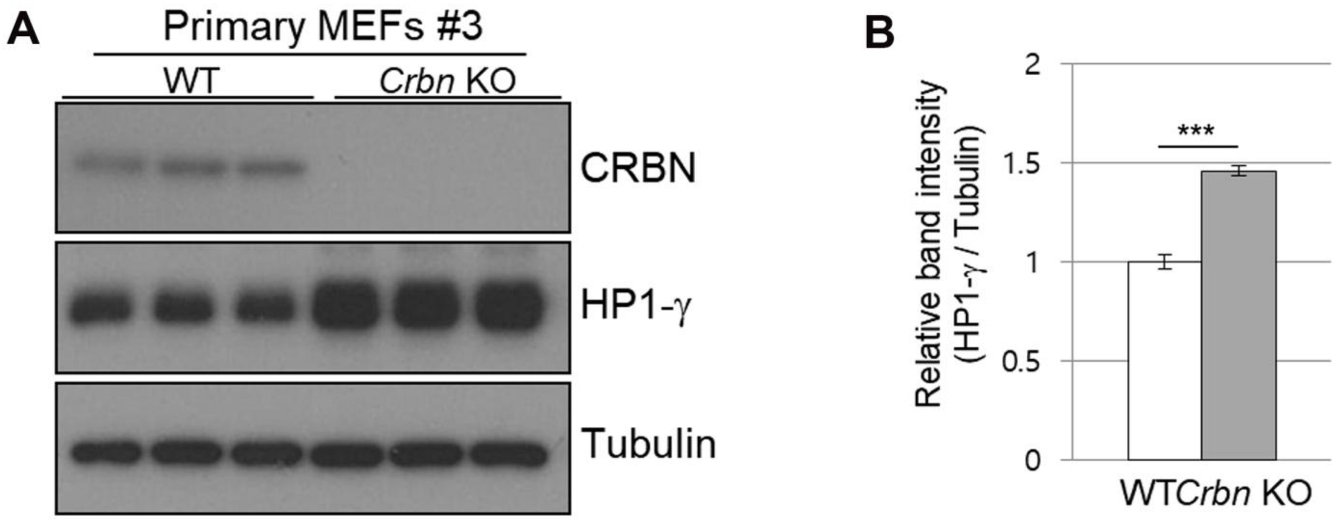

Supplementary Figure 1. The elevated protein level of HP1-γ in primary CRBN KO mouse embryonic fibroblasts. (**A**) Endogenous levels of proteins as determined by Western blot analysis using extracts from WT and CRBN KO MEFs. (**B**) Relative band intensities determined by densitometric analysis of HP1-γ in blot A.

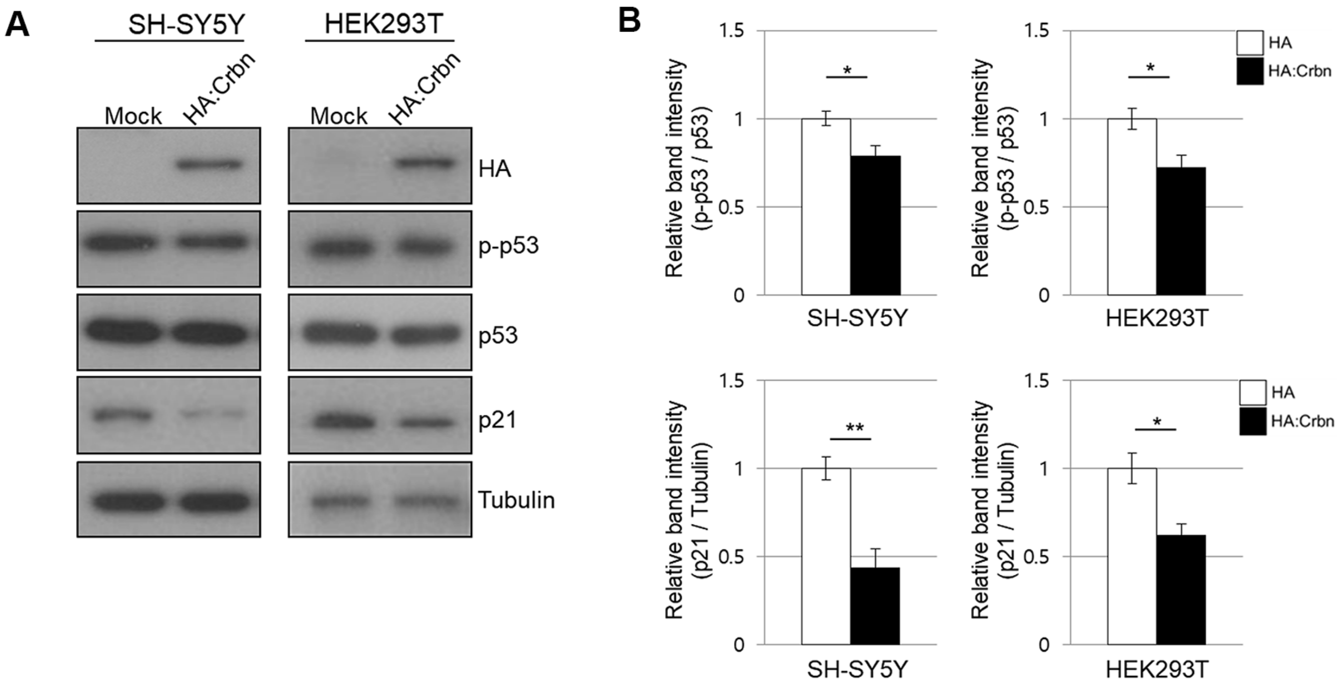

Supplementary Figure 2. Ectopic overexpression of CRBN in SHSY5Y and HEK293T. (**A**) The SHSY5Y and HEK293T cells were transiently transfected with HA:CRBN or empty vector. Cells were harvested after 24h and the protein lysates were subjected to immunoblotting with the anti-HA, anti-p53, anti-p-p53 (Ser18), anti-p21, and anti–Tubulin antibodies. (**B**) Relative band intensities determined by densitometric analysis of each protein in blot A.
